# New Parvovirus and Other Swab-Associated Viruses Identified in the Eurasian Goshawk (*Astur gentilis*)

**DOI:** 10.3390/pathogens15090942

**Published:** 2026-09-05

**Authors:** Agata Prątnicka, Simona Kraberger, Arvind Varsani, Tomasz Stenzel

**Affiliations:** 1 Department of Poultry Diseases, Faculty of Veterinary Medicine, University of Warmia and Mazury in Olsztyn, 10-719 Olsztyn, Poland; 2 Biodesign Center for Fundamental and Applied Microbiomics, Center for Evolution and Medicine, School of Life Sciences, Arizona State University, Tempe, AZ 85287, USA; simona.kraberger@asu.edu (S.K.); arvind.varsani@asu.edu (A.V.); 3 Structural Biology Research Unit, Department of Integrative Biomedical Sciences, University of Cape Town, Observatory, Cape Town 7935, South Africa

**Keywords:** Accipitridae, *Parvoviridae*, *Aveparvovirus*, goshawk, *Astur gentilis*

## Abstract

Cloacal swabs were collected from 12 Eurasian goshawk chicks during routine veterinary inspections to evaluate the gut-associated virome of goshawks used for falconry. The material was used for metagenomic screening with high-throughput sequencing. Sequences belonging to several viral families (*Poxviridae*, *Circoviridae*, *Anelloviridae*, *Retroviridae*, *Parvoviridae* and *Astroviridae*) were detected and their occurrence differed across samples G1-G12. Most of the identified sequences were highly similar to those of viruses that infect poultry, suggesting a probable alimentary transmission route. Two complete genomes of closely related chicken astroviruses, most likely strains belonging to subgroup Biii, were identified. Their ORF2 proteins shared 98.78% amino acid identity. Another complete genome was identified as that of pigeon circovirus with 98.96% nucleotide identity to a strain previously reported from China. A parvovirus genome sequence, distinct from those of known members of the genus *Aveparvovirus* genus was also identified. This parvovirus genome sequence represents approximately 86% of the average aveparvovirus genome length (5422 bp) and contains three open reading frames. The sequence lacks the 3′ region of the capsid gene is missing and the 3′ UTR. The NS1 protein sequence of this virus shares 52.42% amino acid identity with that of its closest relative, the nonstructural protein of silver pheasant parvovirus. These findings suggest that parvovirus infection may occur in birds of prey and that the detected virus may represent a novel member of the genus *Aveparvovirus.* The infected bird showed no clinical symptoms during sampling; thus, the role of this parvovirus in raptor pathology as well as the possibility that it was of dietary origin remains unknown and requires further investigation.

## 1. Introduction

The Eurasian goshawk (*Astur gentilis*), an efficient hunter of game mammals (e.g., rabbits) and game birds (e.g., pheasants, ducks), is one of the species most commonly used for falconry in Poland. In captivity conditions, birds of prey are typically fed a diet consisting of raw day-old chicks, poultry offal (chicken and turkey), quail, pigeons, and portions of hunted game [1,2]. As apex predators, raptors occupy the highest trophic levels and may act as reservoirs and mixing vessels for various pathogens introduced through prey species [3,4].

To date, several viruses have been reported in members of the Accipitridae, including avian influenza virus [5], Newcastle disease virus [6], West Nile virus [7], and, more recently, raptor adenovirus 1 [8,9,10]. Over the last two years, Polish falconers have reported increased mortality among juvenile goshawks during the breeding season, raising concerns about potential infectious causes. These observations highlight the need for systematic investigation of viral agents that may be transmitted via diet or close parental contact.

Parvoviruses are among the smallest known DNA viruses and possess a single-stranded genome of approximately 4–6 kb containing two primary open reading frames (ORFs). The non-structural (NS) region encodes proteins involved in viral replication. Notably, the NS gene is highly conserved across parvoviruses. The structural (VP) region encodes capsid proteins [11]. The family *Parvoviridae* is divided into two subfamilies, *Parvovirinae* and *Densovirinae*, which differ primarily in their host range and infect vertebrates and invertebrates, respectively [11]. The genus *Aveparvovirus* is classified within the subfamily *Parvovirinae* and has been described in various avian hosts, including rock pigeon [12], domestic turkey [13], red-crowned crane [14], pileated finch [15], grass parrot [16], and silver pheasant [17].

Avian parvoviruses are commonly linked to gastrointestinal disorders, particularly in young birds, and the severity of clinical presentation varies with age [18]. Infections with these viruses are frequently associated with clinical signs such as watery diarrhea and growth retardation. In chickens, chicken parvovirus (ChPV) has been implicated in many syndromes, including malabsorption syndrome (MAS)/runting-stunting syndrome (RSS) [19]. These syndromes are commonly associated with diarrhea, poor feed conversion, and impaired growth and development [20]. However, because ChPV genomes have also been identified in clinically healthy birds [12,21], the exact role of the virus in disease development remains uncertain. In recent years, advances in molecular diagnostics and high-throughput sequencing (HTS) technologies have significantly increased the detection and characterization of novel parvoviruses in both healthy and diseased animals [22,23].

Members of the family *Astroviridae* have been identified in a broad range of mammalian (genus *Mamastrovirus*) as well as avian (genus *Avastroviruses*) hosts. The astrovirus genome consists of three open reading frames: ORF1a and ORF1b, which are involved in viral replication, and ORF2, which encodes the capsid protein. Currently, the species classification of avastroviruses is largely based on the host species from which the viruses were originally isolated and is undergoing revision. Based on the amino acid sequence of ORF2, avastroviruses are assigned to one of two major genogroups [24]. Four representative avian astroviruses are currently recognized, including sequences of turkey, duck and chicken origin. Avastroviruses are associated with many clinical symptoms, including embryo mortality [25], growth retardation in chicks [26], white chick disease [27,28], nephritis and hepatic necrosis [29]. Strains have been divided into seven subgroups (Ai, Aii, Aiii, Bi, Bii, Biii and Biv) [30].

Circoviruses have a circular, single-stranded DNA genome of approximately 1.7–2.1 kb. Their genomes typically contain two major ORFs encoding the replication-associated protein (Rep) and the capsid protein (Cap), and the origin of replication is located on the Rep-encoding strand. Circoviruses have been identified in birds, mammals, and freshwater fish [31]. To date, 17 representative avian circovirus species have been identified in various avian hosts. Infections are usually subclinical, but beak and feather disease virus (BFDV) and pigeon circovirus (PiCV) can cause clinical symptoms. BFDV may cause progressive feather abnormalities, beak deformities, and immunosuppression [32], whereas PiCV infection may lead to weight loss, lethargy, diarrhea and other gastrointestinal disorders [33]. The PiCV genome is approximately 2030 bp in length. To date, more than 890 complete PiCV genome sequences have been deposited in the National Center for Biotechnology Information (NCBI) GenBank database. Strains reported in pigeon populations worldwide can be grouped into 13 genotypes [34].

The examined goshawks may be valuable sentinel species for the surveillance of viruses circulating in their prey species. They meet several of the criteria proposed for sentinel species: they are apex predators, falconry birds can be continuously monitored and sampled over time, and they are kept in many countries, allowing for standardized surveillance [35]. Previous studies in other predator species have shown that viruses originating from prey can be detected in fecal samples [36,37] or cloacal swabs [38]. The identification of prey-derived viruses is particularly straightforward when predators feed on taxonomically distant prey, such as birds consuming mammals [37] or fish [38], allowing the origin of the detected genetic material to be determined with greater confidence. In predator-based surveillance, the detection of virus sequence may be opportunistic but nevertheless informative. Occasionally, it may lead to the first detections of a virus in a particular geographic region, as demonstrated by the first mastrevirus reported in the Caribbean, identified through the analysis of dragonflies preying on herbivorous insects [39]. Such findings may provide additional information on viral circulation. At the same time, the results of the present study provide only partial evidence of goshawk usefulness as sentinel species due to the small sample size. Therefore, further research is needed to confirm their reliability as sentinel species.

The aim of the study was to use an HTS approach to investigate the occurrence of viruses potentially transmitted through the alimentary route in juvenile goshawks raised by their parents, while also taking into account close physical contact within the nest environment.

## 2. Materials and Methods

With owners’ consent, samples were collected during routine veterinary visits as part of standard clinical examination. All procedures were performed in accordance with standard animal health and welfare practices. Therefore, approval from the Local Ethics Committee was not required.

### 2.1. Samples

**Figure 1:**
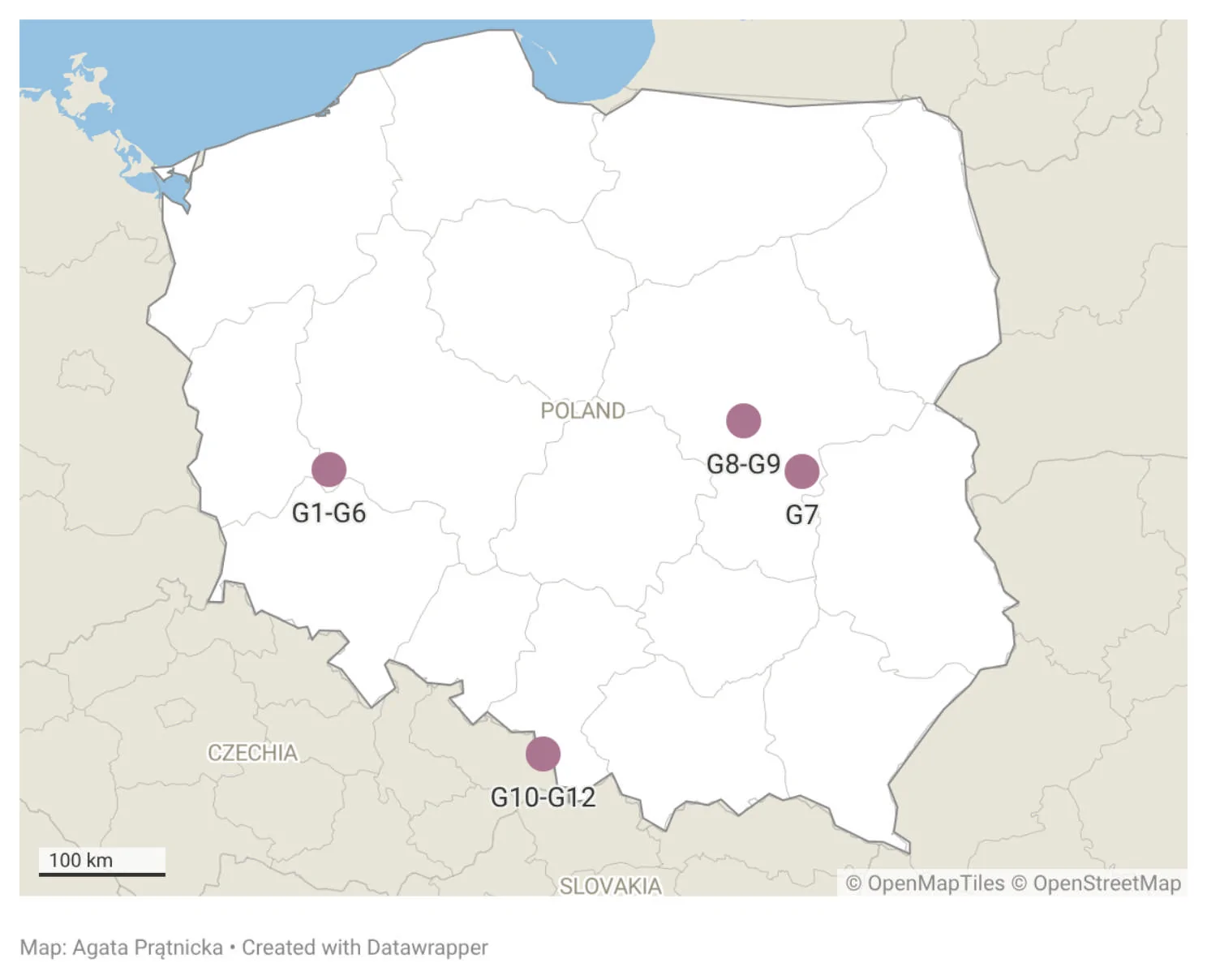
Geographic locations of four sites in Poland where cloacal swabs were collected from Eurasian goshawks (*Astur gentilis*).

Cloacal swabs were collected from 12 Eurasian goshawks aged 4 to12 weeks at four distinct locations between June and August 2025 using swab kits containing universal transport medium (UTM) for viruses, Mycoplasma, and Ureaplasma (Aptaca, Canelli, Italy). The locations of four cloacal sampling sites are shown in Figure 1. The map was created using Datawrapper (Datawrapper GmbH, Berlin, Germany).

Swabs collected on site were transported under cooled conditions in insulated containers to maintain sample integrity. Upon arrival, the samples were immediately processed or stored at −80 °C for long-term preservation.

Viral DNA and total RNA were extracted from 200 µL of UTM buffer. DNA was extracted using the High Pure Viral Nucleic Acid kit (Roche Life Sciences, Pleasanton, CA, USA), and RNA was extracted using the QIAwave RNA Plus Mini Kit (Qiagen, Germantown, MD, USA). Circular DNA molecules were enriched by rolling circle amplification (RCA), using 2 µL of extracted viral DNA as a template with the phi29 DNA Polymerase Kit (Watchmaker Genomics, Boulder, CO, USA). For each DNA sample, 15 µL of the RCA product was combined with 15 µL of the original viral DNA extract, and the resulting mixture was used to prepare high-throughput sequencing libraries with the Illumina DNA LP (M) Tag 96 Sample Preparation Kit (Illumina, San Diego, CA, USA). For RNA, 11 µL of extracted viral RNA was used to generate libraries with the Illumina Stranded Total RNA Prep Kit (Illumina, USA). DNA and RNA libraries were sequenced (2 × 150 bp, paired-end) in multiplex mode on an Illumina NovaSeq X sequencer at Psomagen Inc. (Rockville, MD, USA).

### 2.2. De Novo Assembly of Viral Genomes

Raw sequencing reads were quality-trimmed at the 5′ and 3′ ends using Trimmomatic v 0.39 [40] with default settings. Trimmed reads were de novo assembled into contigs using MEGAHIT v.1.2.9 [41]. Contigs exceeding >1000 nucleotides were analyzed for virus-like sequences using Diamond BLASTx [42] against the Viral RefSeq protein sequence database (release 230) and Cenote-Taker3 [43].

Reads originating from the host were not removed or depleted prior to further analysis, as the analyzed material consisted of cloacal swabs, which inherently contain reads from both the host and the associated bacterial communities. To assess potential cross-contamination between libraries, reads from each library were mapped using CoverM [44] against contigs obtained from the de novo assembly of data from the corresponding library. No evidence of cross-contamination between libraries was found. In addition, negative controls were included at all stages of the experimental and sequencing procedures to monitor for potential sample contamination.

### 2.3. Sequence Analysis

Coding-complete viral genomes were annotated using Geneious Prime 2026.1.1 (Dotmatics, Boston, MA, USA), including the identification of ORFs and coding sequences. The annotated genomes were used to construct a reference dataset of viral sequences within the corresponding taxonomic groups using sequences retrieved from GenBank.

The alimentary-transmitted viruses detected in this study were subjected to basic phylogenetic analysis according to the recommendations of the International Committee on Taxonomy of Viruses (ICTV) [45]. Sequence similarity analysis was performed using the ORF2 protein for astroviruses and the complete genome nucleotide sequence for the circovirus. BLASTn and BLASTp were used accordingly to identify the closest matching sequences available in the GenBank database. For astroviruses, the four most similar chicken astrovirus sequences with 100% query coverage and >80% sequence identity were selected for analysis, together with one more distantly related chicken astrovirus strain for comparison, representative avastrovirus sequences from other species identified in the BLAST search and ICTV reference sequences. For the circovirus, in addition to the closest 14 matches identified by BLASTn, representative strains originating from different continents were also included in the analysis. The GenBank accession numbers and strain names of viruses used in the analysis are presented in Supplementary Table S1.

The putative novel parvovirus sequence was analyzed as follows. According to the species demarcation criteria established by the ICTV *Parvoviridae* study group [11], the ORF encoding the NS1 protein was extracted and translated into the corresponding amino acid sequence using Geneious Prime. Similar sequences were identified using a BLASTp search against the NCBI database. Sequences with comparable protein lengths (>500 amino acids) were selected and downloaded for bioinformatic analyses. In addition, NS1 sequences representing the 11 aveparvoviruses (MW588065, OP894050, KC876004, GU214706, MT138241, KY312546, MG745672, MT138209, MT138299, MW046460, MT138266) recognized by ICTV were included, together with bovine parvovirus-1 (DQ335247), which was used as an outgroup for tree rooting. Reference sequences and the sequences of the closest parvoviruses identified by BLASTp analysis used in this analysis are listed in Supplementary Table S2 [12,14,15,17,23,46,47,48,49,50,51,52].

Multiple sequence alignment was performed using MAFFT [53] implemented in Geneious Prime (Dotmatics, USA). The resulting alignment was trimmed using trimAl [54] to remove poorly aligned regions. The curated alignment was subsequently analyzed in MEGA12 [55] to determine the best-fit amino acid substitution model based on the Bayesian Information Criterion (BIC). LG+G+I+F was selected as the optimal model for the astrovirus and parvovirus sequence datasets, whereas GTR+G+I was selected for circovirus sequences. A maximum likelihood phylogenetic tree was then generated using IQ-TREE3 [56]. The resulting phylogenetic tree was visualized with iTol [57]. Pairwise sequence similarity matrices were generated using SDT v1.3 [58], and sequence alignments were calculated using the MUSCLE algorithm [59].

## 3. Results and Discussion

### 3.1. Known Viruses

Virus sequences were identified in 11/12 (91.7%) birds. However, only three sequences (two chicken astroviruses and one PiCV) represented complete genomes. *Astroviridae* sequences were found in 2/12 (16.7%) birds and were most likely of dietary origin given their high similarity to those from chickens (>92% sequence similarity in both cases). Sequences belonging to members of the family *Poxviridae* were identified in 11/12 (91.7%) birds, including fowlpox virus in 8/12 (66.7%) birds, turkeypox virus in 7/12 (58.3%) birds, and pigeonpox virus in 2/12 (16.7%) birds. More than one member of the family *Poxviridae* was detected in 4/12 (33.3%) birds. In raptors, cases of avipoxvirus infection have been reported in both Falconiformes, including peregrine falcons, saker falcons, gyrfalcons, and falcon hybrids [60,61], and Accipitriformes, such as goshawks [62], white-tailed eagles [63], and turkey vultures [64]. Yet, there are no direct reports in the literature describing the occurrence of avipoxviruses in the digestive tract of raptors. In previously published case reports, diagnoses were generally based on the evaluation of dry cutaneous lesions [60], PCR [63], and Bollinger bodies identified in a histopathological examination [61].

Pigeon circovirus (PiCV) was identified in 2/12 (16.7%) birds. Similar findings were reported in cloacal samples from clinically healthy juvenile crested caracaras (*Caracara plancus*) [65], juvenile black vultures (*Coragyps atratus*) [65], and peregrine falcons (*Falco peregrinus*) [66], suggesting possible passive ingestion. The presence of PiCV genetic material in 4-week-old goshawk chicks examined in this study suggests that pigeons may have been used as feed for those birds.

Avian gyrovirus 2, a member of the family *Anelloviridae*, was identified in 1/12 (8.3%) birds. This virus has been widely detected in chickens, including in organ and serum samples [67,68,69], and chickens are therefore considered its primary host. Although the presence of avian gyrovirus 2 has been confirmed in predator tissues, such as snake liver, the route of cross-species transmission remains unknown, and it is uncertain whether the virus originates from chickens used as feed [70]. The occurrence of gyrovirus in raptor samples is likely related to their poultry-based diet, although cross-species transmission cannot be excluded.

A representative of the family *Retroviridae*, identified as Reticuloendotheliosis virus, was detected in 2/12 (16.7%) birds in this study. Reticuloendotheliosis virus has been described in both commercial poultry [71] and, less frequently, in wild birds such as pheasants [72]. It is well recognized for inducing neoplastic lesions [73] and immunosuppression [74] in chickens. In recent years, Reticuloendotheliosis virus has also been confirmed as the causative agent of liver neoplasia in Muscovy ducks [75] and tumor-like lesions affecting the lungs, heart and liver of pigeons [76]. To date, no data on birds of prey have been published for comparison with our results. Based on the previously reported host species, Reticuloendotheliosis virus identified in goshawk samples is likely of dietary origin. Members of the family *Parvoviridae* were detected in 2/12 (16.7%) birds, including a novel aveparvovirus identified in bird G8.

Although percentage values are reported above, the prevalence or viral distribution cannot be reliably estimated due to the small sample size used in this study. It should also be noted that the food provided to the goshawks and their tissues were not analyzed as part of this study; therefore, the origin of the viruses cannot be definitively determined. The viral genetic material identified in the investigated samples is listed in Table 1. The sequencing results for bird G12 consisted exclusively of phages.

The number of mapped reads and mean read depth for the samples: G3, G5, G7, and G8 were determined using coverM [44]. The results are presented in Table 2.

### 3.2. Bioinformatic Analysis of Complete Genomes

#### 3.2.1. Astrovirus

**Figure 2:**
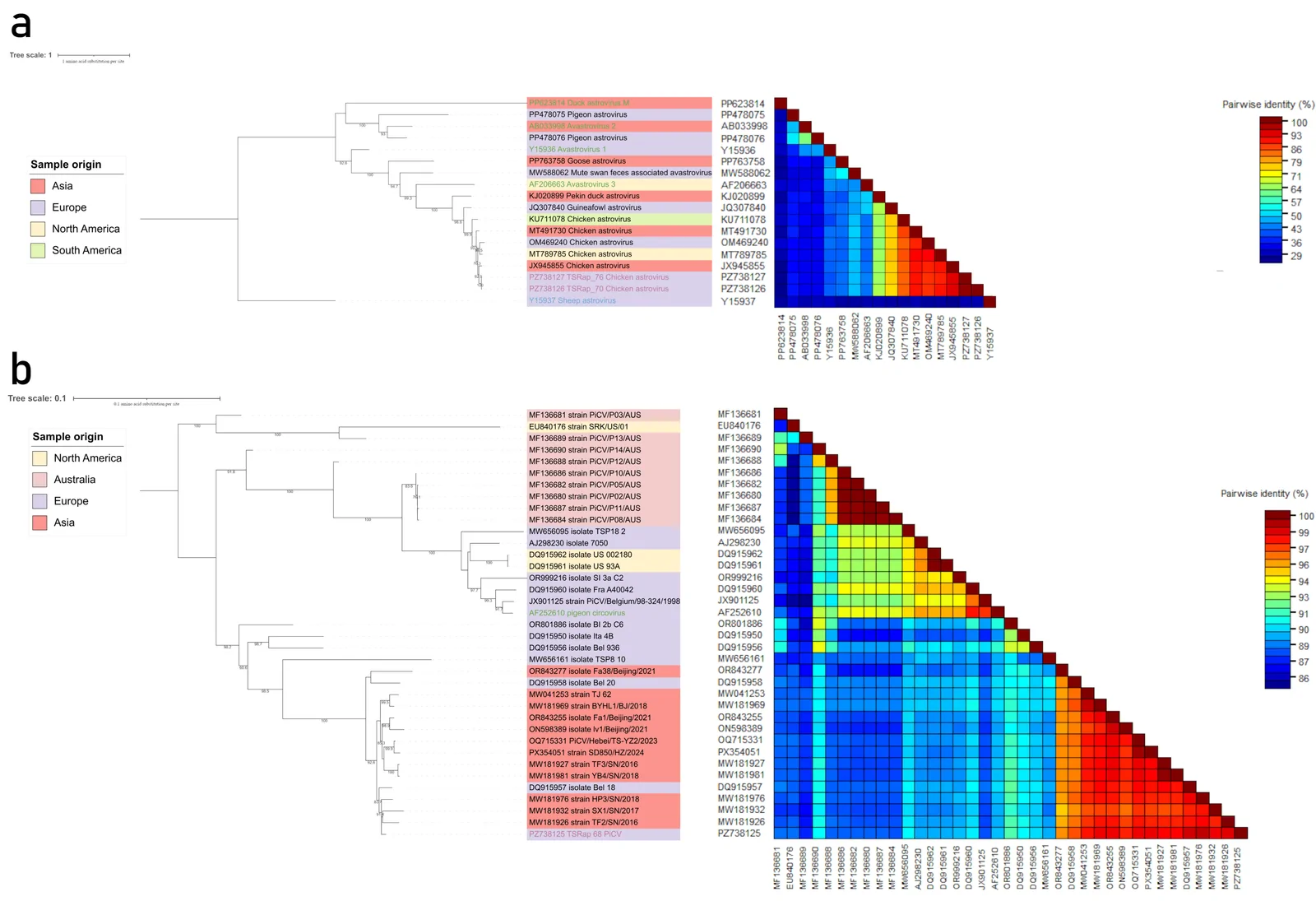
(**a**) Phylogenetic analysis of chicken astrovirus identified in goshawk cloacal swabs: maximum likelihood phylogenetic tree (left) and pairwise identity matrix of amino acid sequences of capsid protein sequences (right). Branch support was assessed with 1000 ultrafast bootstrap replicates and 1000 SH-aLRT replicates. The analysis included 18 capsid protein amino-acid sequences: two sequences identified in this study (purple font), four reference avastrovirus sequences (green font) and 11 avastrovirus sequences from chickens and other host species representing the closest (BLASTp) matches (black font). After trimming the sequences, the alignment length was 645 amino acids, of which 523 sites were parsimony-informative. The sheep astrovirus was used for rooting the tree (blue font) (**b**) Phylogenetic analysis of pigeon circovirus identified in a goshawk cloacal swab: maximum likelihood phylogenetic tree (left) and genome-wide nucleotide sequence similarity matrix generated using the same set of sequences (right). Branch support was evaluated using 1000 ultrafast bootstrap replicates and 1000 SH-aLRT replicates. The complete nucleotide sequence identified in the goshawk sample (purple font) was compared with the pigeon circovirus reference sequence (green font) and other 35 complete-genome circoviral strains recovered worldwide (black font color corresponding to the legend). After trimming the sequences, the alignment length was 2041 nucleotides, of which 501 sites were parsimony-informative.

Two astrovirus genomes were identified, comprising 7502 nt in bird G5 and 7487 nt in bird G7. The genome sequences of these viruses were submitted to the GenBank (NCBI) database under accession numbers PZ738126 and PZ738127, respectively. The ORF2 gene (capsid protein gene) in both sequences encodes a protein composed of 738 amino acids. The ORF2 amino acid sequences from these two samples share 98.78% similarity, with differences at nine amino acid positions: 32 (G5: N, G7:S), 42 (G5: E, G7: D), 391 (G5: T, G7:A), 416 (G5: S, G7: G), 424 (G5: V, G7: I), 557 (G5: A, G7: T), 605 (G5: I, G7: V), 711 (G5: N, G7: T) and 724 (G5: L, G7: P). In both cases, the most similar sequence identified by BLASTp was chicken avastrovirus (JX945855), which shares 92.28% and 92.55% similarity with PZ738126 and PZ738127, respectively (Figure 2a). This sequence belongs to subgroup Biii and represents a strain reported to be associated with renal disease and visceral gout in domestic chicken [29]. Approximately 88% sequence identity was observed with a Canadian strain (MT789785) associated with white chick syndrome [27].

#### 3.2.2. Circovirus

A complete circoviral genome (2032 nt; accession No. PZ738125; isolate: TSRap_68) was identified in bird G3. According to the ICTV, species demarcation for circoviruses is based on genome-wide nucleotide sequence identity and the distribution of pairwise sequence identities [77]. The complete genome sequence of this virus shared 98.96% nucleotide identity with its most related strain MW181976. Phylogenetic analysis demonstrated that the PiCV sequence (PZ738125) clustered more closely with circovirus sequences recovered from Chinese pigeons [78] than with the previously reported Polish PiCV isolates [79] (Figure 2b).

### 3.3. Novel Goshawk Parvovirus

Although a fragment of the parvovirus sequence was successfully sequenced in birds G7, it was too short (267 bp) for a detailed analysis. An incomplete parvovirus genome of 4638 nt, representing approximately 86% of the average *Aveparvovirus* genome length (4638/5422 = 85.5%), was identified in bird G8. The genome sequence of this virus was submitted to the GenBank (NCBI) database under accession number PZ600707. The genome architecture of the virus follows the typical parvovirus organization, with ORFs encoding NS and VP, as well as an additional accessory ORF between these regions [80]. The NS1 gene consists of 1870 bp (positions 60–1929), the hypothetical protein gene consists of 916 bp (positions 1972–2887), and the partial capsid protein gene consists of 1778 nt (positions 2861–4638). The hypothetical protein gene is located in the middle of the genome and shares 27 nucleotides with the capsid protein, as their ORFs overlap. The NS1 protein shares only 52.42% amino acid sequence identity with the nonstructural protein of Lophura nycthemera aveparvovirus (QTE03934.1) from a silver pheasant [17], its closest relative identified by BLASTp analysis. The NS1 protein contains a helicase, with a conserved P-loop (Walker A) motif that mediates ATP or GTP binding, GxxxxGKS/T (_402_**G**PANT**GKT**_409_) and approximately 33 amino acids downstream, a Walker B-like motif, xxxxEE (_441_LIWW**EE**_447_). This motif is located just downstream of the predicted AAA+ ATPase superfamily domain, xLxxxxxxxxYGPxxTGKTxxAxxIAxxxxxxG (_391_K**L**GKKNTIYF**YGP**AN**TGKT**MM**A**ES**IA**KMIKLF**G**_423_). NS1 also encodes a conserved replication initiator (endonuclease) motif, xxHuHxxxx (KV_122_**H**I**H**_124_IIFI), responsible for nicking viral DNA. Conserved domain annotations were identified using NCBI’s Conserved Domain Search tool [81] against the CDD database [82].

To date, a parvovirus in birds of prey has been described only in the barn owl [83]. However, the genome structure of barn owl parvovirus differs from that of the virus identified in this study (with four ORFs: NS1, NS2, VP1, and VP2), and this virus was classified in the genus *Chaphamaparvovirus* within the subfamily *Hamaparvovirinae*.

According to the species demarcation criteria established by the ICTV *Parvoviridae* study group [11], parvoviruses are considered to belong to the same species when their NS1 proteins share more than 85% amino acid sequence identity [84]. The comparative analysis of the NS1 sequence of the parvovirus identified in this study with comparable NS1 sequences from other known avian parvoviruses deposited in the GenBank database indicates that it represents a new species based on its 52.42% amino acid sequence identity with the closest relative. Under the proposed demarcation criteria, NS1 proteins from members of the same genus are expected to share ≥35–40% amino acid sequence identity, with >80% coverage in pairwise comparisons [85]. In this case, the pairwise BLAST analysis reveals 41.59–53.02% amino acid sequence identity with members of the genus Aveparvovirus, with query coverage ranging from 86% to 100%. Phylogenetic analysis showed that the NS1 protein of the novel parvovirus clustered with the previously described aveparvoviruses and represented a clearly distinct lineage.

**Figure 3:**
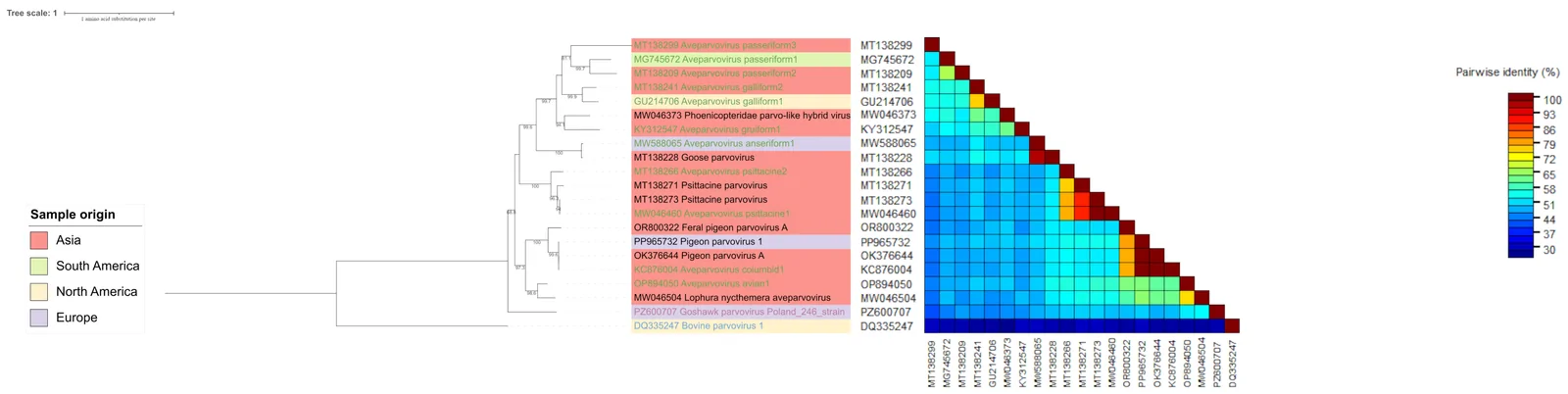
Phylogenetic analysis of the novel goshawk parvovirus identified in this study. The amino acid sequence of NS1 of the novel aveparvovirus was compared with ICTV reference aveparvovirus sequences, and the most similar sequences were identified using the Basic Local Alignment Search Tool (BLAST). These sequences were retrieved from the GenBank database. Branch support was assessed using 1000 ultrafast bootstrap replicates and 1000 SH-aLRT replicates. The novel parvovirus is marked in purple font, reference viruses are shown in green font, the non-reference viruses identified as similar by BLAST are written in black font and the outgroup strain is highlighted in blue font. After trimming the sequence, the alignment length was 499 amino acids, of which 330 sites were parsimony-informative. The pairwise identity matrix of amino acid sequences for the non-structural protein (NS1) of goshawk aveparvovirus and sequences retrieved from the GenBank database, generated using the Sequence Demarcation Tool (SDT), are shown on the right. All sequences are labeled with their accession numbers. Amino acid identity between each pair of sequences is displayed in the matrix, with colors indicating percentage identity values, ranging from dark blue for the lowest identity to dark red for the highest identity.

**Figure 4:**
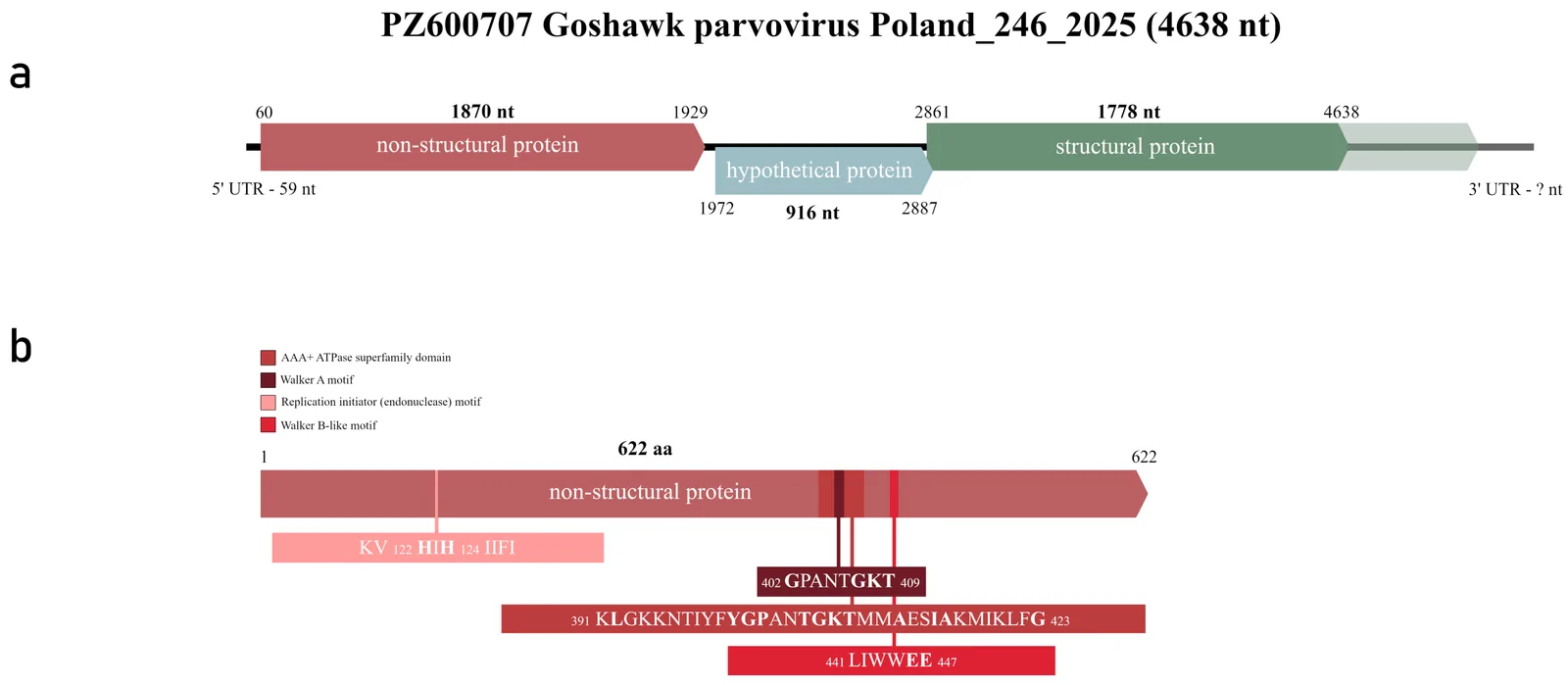
(**a**) Schematic diagram of the genome organization of the goshawk parvovirus sequence obtained in this study. The structural protein is incomplete, representing approximately ~85% length of the closest available reference sequence (pigeon parvovirus A, KC876004, species *Aveparvovirus columbid1*). (**b**) Organization of the NS1 protein showing the positions of conserved domains.

The phylogenetic tree and the pairwise similarity matrix are presented in Figure 3. The genome organization of goshawk aveparvovirus is shown in Figure 4.

### 3.4. Clinical Findings

Samples were collected from asymptomatic birds. The bird in which the parvovirus was detected died approximately three months after sample collection (October 2025) and was reported to be in poor body condition (cachectic), with starvation suspected as the probable cause of death. According to the owner obstruction of the proventriculus by food was suspected. The bird also exhibited decreased vitality during flight and general lethargy. No necropsy was performed because the owner did not make the carcass available for examination. Poor body condition at the time of death may be consistent with the symptoms observed in other birds infected with parvoviruses [86,87], although a possible association with parvovirus infection cannot be determined in this case.

## 4. Conclusions

Viral analysis of cloacal swabs collected from several-week-old goshawks showing no clinical signs at the time of sampling revealed the presence of representatives of six viral families. These viruses are likely of dietary origin, but their origin could not be definitively confirmed as food samples, and the goshawk tissues were not examined. The newly identified parvovirus appears to be highly divergent from the currently known aveparvoviruses, and the partial genome obtained indicates the need for further studies to determine its genomic organization and relationship to other known viruses. As no tissue samples were available for analysis, it was not possible to determine whether the detected virus represented an active infection or was of dietary origin.

## Figures and Tables

**Table 1:** Virological findings in cloacal swabs from juvenile goshawks.

Sample #	Age	Virus Detected	Complete Genome Recovered	Contig Length [nt]	No. of Reads per Sample
G1	8 weeks	*Poxviridae*:			42,970,834
		Fowlpox virus	No	756	
		Turkeypox virus	No	699	
G2	4 weeks	*Circoviridae*:			50,045,262
		Pigeon circovirus	No	333	
		*Poxviridae*:			
		Turkeypox virus	No	264	
G3	4 weeks	*Circoviridae*:			39,046,362
		Pigeon circovirus	Yes ^1^	2032	
		*Anelloviridae*:			
		Avian gyrovirus 2	No	297	
		*Poxviridae*:			
		Fowlpox virus	No	456	
		*Retroviridae*:			
		Reticuloendotheliosis virus	No	243	
G4	7 weeks	*Poxviridae*:			715,136
		Fowlpox virus	No	327	
G5	7 weeks	*Poxviridae*:			45,724,662
		Turkeypox virus	No	876	
		Fowlpox virus	No	474	
		Pigeonpox virus	No	591	
		*Astroviridae*:			
		Chicken astrovirus	Yes ^2^	7502	
G6	7 weeks	*Poxviridae*:			40,469,300
		Fowlpox virus	No	588	
		Pigeonpox virus	No	507	
		Turkeypox virus	No	264	
G7	12 weeks	*Parvoviridae*:			41,325,720
		Pigeon parvovirus A	No	267	
		*Poxviridae*:			
		Fowlpox virus	No	522	
		*Astroviridae*:			
		Chicken astrovirus	Yes ^3^	7487	
G8	9 weeks	*Parvoviridae*:			54,127,704
		Pigeon parvovirus A	No *	4638	
		*Poxviridae*:			
		Turkeypox virus	No	726	
		*Retroviridae*:			
		Reticuloendotheliosis virus	No	1683	
G9	9 weeks	*Poxviridae*:			62,909,706
		Fowlpox virus	No	270	
		Turkeypox virus	No	294	
G10	9 weeks	*Poxviridae*:			28,501,212
		Turkeypox virus	No	321	
G11	9 weeks	*Poxviridae*:			47,607,508
		Fowlpox virus	No	540	
G12	10 weeks	N/A	N/A	N/A	29,456,492

^1^ Strain: TSRap_68; Accession No. PZ738125. ^2^ Strain: TSRap_70; Accession No. PZ738126. ^3^ Strain: TSRap_76; Accession No. PZ738127. * Strain: Poland_246_2025; Accession No. PZ600707. N/A—not applicable.

**Table 2:** Mean read depth and the number of mapped reads for selected samples.

Sample #; Virus Sequence Accession #	Number of Mapped Reads	Mean Read Depth
G3; PZ738125	490	38.04
G5; PZ738126	2190	44.33
G7; PZ738127	668	13.58
G8; PZ600707	442	15.08

## Data Availability

Sequencing data were deposited in the NCBI Sequence Read Archive under the following accession numbers BioProject #No. PRJNA1482152; BioSample #Nos. SAMN61114219, SAMN61478120, SAMN61478121, SAMN61478122, SAMN61478123, SAMN61478124, SAMN61478125, SAMN61478126, SAMN61478127, SAMN61478128, SAMN61478129, SAMN61478130; SRA #Nos. SRR39326663, SRR39526555, SRR39526556, SRR39526557, SRR39526561, SRR39526562, SRR39526563, SRR39526564, SRR39526565, SRR40336053, SRR40336054, SRR40362984, SRR40362985, SRR40396567. The parvovirus sequence was deposited in GenBank under accession #No. PZ600707. The circovirus sequence was deposited in GenBank under accession #No. PZ738125. Astrovirus sequences were deposited in GenBank under accession #Nos. PZ738126 and PZ738127.

## Supplementary Materials

The following supporting information can be downloaded at: https://www.mdpi.com/article/10.3390/pathogens15090942/s1, Table S1: Viral strains and corresponding sequence accession numbers used for the comparative analysis; Table S2: The parvovirus sequences used for comparison in this study.
