# Supplementary material for: New Parvovirus and Other Swab-Associated Viruses Identified in the Eurasian Goshawk (Astur gentilis)
Source: Pathogens. 2026 Sep 5;15(9):942. doi: 10.3390/pathogens15090942 (PMC13610506; doi:10.3390/pathogens15090942)
Supplement: Supplementary file 1 [file pathogens-15-00942-s001.zip › Table S2.pdf]

**Table S2. The parvovirus sequences used for comparison in this study**

| Virus species / name | Host | Genome length | NS1 length | Accession number | References |
|---|---|---|---|---|---|
| *Aveparvovirus anseriform1* | Mute swan | 5717 bp | 622 aa | MW588065 | [1] |
| *Aveparvovirus avian1* | Blue peacock, red-waisted golden pheasant, Reeve's pheasant, silver pheasant, cockatoo, grey crane, white-naped crane | 5909 bp | 639 aa | OP894050 | [2] |
| *Aveparvovirus columbid1* | Rock pigeon | 5492 bp | 631 aa | KC876004 | [3] |
| *Aveparvovirus galliform1* | Turkey | 4615 bp | 694 aa | GU214706 | [4] |
| *Aveparvovirus galliform2* | Golden pheasant | 5291 bp | 612 aa | MT138241 | [5] |
| *Aveparvovirus gruiform1* | Red-crowned crane | 5515 bp | 680 aa | KY312546 | [6, 7] |
| *Aveparvovirus passeriform1* | Pileated finch | 5368 bp | 612 aa | MG745672 | [8] |
| *Aveparvovirus passeriform2* | The black-faced bunting | 5208 bp | 620 aa | MT138209 | [5] |
| *Aveparvovirus passeriform3* | Radde's warbler | 5056 bp | 624 aa | MT138299 | [5] |
| *Aveparvovirus psittacine1* | Blue-and-yellow macaw | 5458 bp | 632 aa | MW046460 | [9] |
| *Aveparvovirus psittacine2* | Mealy amazon | 6015 bp | 682 aa | MT138266 | [5] |
| Phoenicopteridae parvo-like hybrid virus | Phoenicopteridae | 2977 bp | 615 aa | MW046373 | [9] |
| Goose parvovirus | Greater White-fronted Goose | 4868 bp | 539 aa | MT138228 | [5] |

| | | | | | |
|---|---|---|---|---|---|
| Psittacine parvovirus | Psittacidae | 5702 bp | 635 aa | MT138271 | [5] |
| Psittacine parvovirus | Red-fan parrot | 5684 bp | 632 aa | MT138273 | [5] |
| Lophura nycthemera aveparvovirus | Silver pheasant | 2285 bp | 649 aa | MW046504 | [9] |
| Feral pigeon parvovirus A | Feral pigeon | 3695 bp | 626 aa | OR800322 | Unpublished |
| Pigeon parvovirus 1 strain USA | Feral pigeon | 5128 bp | 566 aa | PP965732 | [10] |
| Pigeon parvovirus A strain FJ01 | Racing pigeon | 1896 bp | 631 aa | OK376644 | [11] |
| Bovine parvovirus-1 strain Abinanti | Cow | 5515 bp | 860 aa | DQ335247 | [12] |
| Eurasian goshawk parvovirus | Eurasian goshawk | 4638 bp | 622 aa | PZ600707 | This study |


1. Hill, S.C., et al., *Impact of host age on viral and bacterial communities in a waterbird population.* Isme j, 2023. **17**(2): p. 215-226.
2. Wang, Y., et al., *Molecular detection and characterization of three novel parvoviruses belonging to two different subfamilies in zoo birds.* Arch Virol, 2023. **168**(6): p. 163.
3. Phan, T.G., et al., *The viruses of wild pigeon droppings.* PLoS One, 2013. **8**(9): p. e72787.
4. Day, J.M. and L. Zsak, *Determination and analysis of the full-length chicken parvovirus genome.* Virology, 2010. **399**(1): p. 59-64.
5. Shan, T., et al., *Virome in the cloaca of wild and breeding birds revealed a diversity of significant viruses.* Microbiome, 2022. **10**(1): p. 60.
6. Wang, Y., et al., *The fecal virome of red-crowned cranes.* Arch Virol, 2019. **164**(1): p. 3-16.
7. Liu, H., et al., *Isolation and characterization of a novel parvovirus from a red-crowned crane, China, 2021.* BMC Vet Res, 2023. **19**(1): p. 169.
8. de Souza, W.M., et al., *Novel Parvoviruses from Wild and Domestic Animals in Brazil Provide New Insights into Parvovirus Distribution and Diversity.* Viruses, 2018. **10**(4).
9. Dai, Z., et al., *Parvovirus dark matter in the cloaca of wild birds.* Gigascience, 2022. **12**.
10. Łukaszuk, E., et al., *Known and novel parvoviruses identified in domestic pigeons.* BMC Vet Res, 2025. **21**(1): p. 47.
11. Chen, C., et al., *Specific detection of pigeon parvovirus with TaqMan real-time PCR technology.* Poult Sci, 2025. **104**(1): p. 104541.
12. Qiu, J., et al., *The transcription profile of the bocavirus bovine parvovirus is unlike those of previously characterized parvoviruses.* J Virol, 2007. **81**(21): p. 12080-5.
